# Formononetin Ameliorates Renal Dysfunction, Oxidative Stress, Inflammation, and Apoptosis and Upregulates Nrf2/HO-1 Signaling in a Rat Model of Gentamicin-Induced Nephrotoxicity

**DOI:** 10.3389/fphar.2022.916732

**Published:** 2022-05-26

**Authors:** Osama Y. Althunibat, Mohammad H. Abukhalil, Saleem H. Aladaileh, Haitham Qaralleh, Wesam Al-Amarat, Manal A. Alfwuaires, Abdulmohsen I. Algefare, Nader Ibrahim Namazi, Sahar J. Melebary, Ahmad O. Babalghith, Carlos Adam Conte-Junior

**Affiliations:** ^1^ Department of Medical Analysis, Princess Aisha Bint Al-Hussein College of Nursing and Health Sciences, Al-Hussein Bin Talal University, Ma’an, Jordan; ^2^ Department of Biology, College of Science, Al-Hussein Bin Talal University, Ma’an, Jordan; ^3^ Department of Medical Laboratory Sciences, Mutah University, Karak, Jordan; ^4^ Department of Medical Support, Al-karak University College, Al-Balqa’ Applied University, As-Salt, Jordan; ^5^ Department of Biological Sciences, Faculty of Science, King Faisal University, Al-Ahsa, Saudi Arabia; ^6^ Pharmaceutics and Pharmaceutical Technology Department, College of Pharmacy, Taibah University, Al Madinah Al Munawarah, Saudi Arabia; ^7^ Department of Biology, College of Science, University of Jeddah, Jeddah, Saudi Arabia; ^8^ Medical Genetics Department, College of Medicine, Umm al-qura University, Makkah, Saudi Arabia; ^9^ Center for Food Analysis (NAL), Technological Development Support Laboratory (LADETEC), Federal University of Rio de Janeiro (UFRJ), Rio de Janeiro, Brazil

**Keywords:** formononetin, gentamicin, nephrotoxicity, inflammation, oxidative stress, Nrf2

## Abstract

Gentamicin (GEN) is a bactericidal aminoglycoside known to cause nephrotoxicity. Formononetin (FN) is a potent flavonoid that exhibits numerous promising pharmacological activities. In this study, we have assessed the nephroprotective efficacy of FN against GEN-induced renal injury in rats. Rats were orally administered with FN (60 mg/kg/day, for 2 weeks) and were co-treated with intraperitoneal (i.p.) injection of GEN (100 mg/kg/day) during the days 8–14. GEN-treated rats demonstrated increased urea and creatinine levels in serum associated with marked histopathological changes in the kidney. Malondialdehyde (MDA) and protein carbonyl contents were elevated, whereas glutathione concentration and catalase and superoxide dismutase activities were lowered in GEN-administered rats. The FN largely prevented tissue damage, attenuated renal function, reduced MDA and protein carbonyl, and enhanced antioxidant capacity in the kidney of GEN-administrated animals. The kidney of GEN-treated rats demonstrated elevated Bax and caspase-3 protein expression, accompanied by lowered Bcl-2 protein expression, an effect that FN attenuated. Moreover, FN treatment caused upregulation of nuclear factor erythroid 2-related factor 2 (Nrf2) and heme oxygenase 1 (HO-1) expression in renal tissue of GEN-intoxicated animals. Collectively, FN protects against GEN-caused renal damage via exhibiting antioxidant, anti-inflammatory, and antiapoptotic activities and augmenting Nrf2 signaling, suggesting FN as a promising agent for preventing drug-induced organ damage.

## Introduction

Gentamicin (GEN) is a bactericidal aminoglycoside used to treat potentially fatal Gram-negative bacterial infections (Randjelovic et al., 2017; Abdel-Fattah et al., 2021). Unfortunately, the therapeutic application of GEN is frequently restricted by the induction of nephrotoxicity. It has been reported that nephrotoxicity appears in 10–25% of patients after treatment with GEN (Quiros et al., 2011). GEN-induced kidney injury is manifested by necrosis and apoptosis of tubular cells and inflammatory cell infiltration (Balakumar et al., 2010; Quiros et al., 2011; Randjelovic et al., 2017). The primary mechanisms behind the nephrotoxicity of GEN include, but are not limited to, increased free radical (FR) formation and activation of pro-inflammatory and cell death pathways, which eventually culminate in renal dysfunction (Balakumar et al., 2010; Ahmed and Mohamed, 2019; Medić et al., 2019; Abdel-Fattah et al., 2021). Therefore, therapeutic approaches aimed at attenuating oxidative stress (OS) and inflammation can protect against the devastating complication of GEN.

Indeed, excessive reactive oxygen species (ROS) production provokes deleterious cellular effects, including direct damage to lipids, oxidative damage of deoxyribonucleic acid (DNA), and protein oxidation. In addition, GEN-induced ROS overproduction is linked to nuclear factor-kappa B (NF-κB) activation, which results in the activation of several inflammatory components, particularly pro-inflammatory cytokines, eventually culminating in renal cell apoptosis and nephrotoxicity (Balakumar et al., 2010; Randjelovic et al., 2017; Ahmed and Mohamed, 2019; Li et al., 2022). Since OS is an important event in the induction of GEN nephrotoxicity, activation of various antioxidant and cytoprotective enzymes is essential. The nuclear factor erythroid 2-related factor 2 (Nrf2) is among the possible druggable targets that can prevent oxidative tissue injury induced by various redox insults (Satta et al., 2017; El-Sayed et al., 2021). It is a critical coordinator of the cellular stress reaction by modulating the expression of a plethora of cytoprotective and antioxidant enzymes (Shelton et al., 2013; He et al., 2020). Boosting the Nrf2 activity has been shown to attenuate drug-induced oxidative tissue damage in animals (Mahmoud et al., 2017a; Aladaileh et al., 2019a; Aladaileh et al., 2019b; Aladaileh et al., 2021a). Conversely, Nrf2-deficient animals were more vulnerable to a wide spectrum of chemical toxicity and pathological situation (Liu et al., 2009; Klaassen and Reisman, 2010). Hence, activation of Nrf2 might represent a novel therapeutic approach to avert drug-induced tissue injury.

Formononetin (FN) is a natural isoflavone found in several plants, mainly of the “Fabaceae” family (Tay et al., 2019; Dutra et al., 2021). Various scientific reports have demonstrated that FN exhibits antioxidant, anti-inflammatory anti apoptotic, and tissue protective properties (Aladaileh et al., 2019a; Yi et al., 2020). It has been shown that FN improved renal function and demonstrated nephroprotective properties through ameliorating oxidative damage and lowering the degree of pro-inflammatory cytokines *in vivo* and *in vitro* models of cisplatin-induced nephrotoxicity (Shinde et al., 2021). In addition, FN attenuated rhabdomyolysis-induced kidney apoptosis by upregulating Nrf2 (Huang et al., 2016)*.* Moreover, FN protected mice from acetaminophen-induced hepatotoxicity, which was attributed to its ability to activate Nrf2 (Jin et al., 2017). Furthermore, a recent study showed that FN upregulated Nrf2/heme oxygenase 1 (HO-1) signaling and prevented renal oxidative injury, inflammatory reaction, and apoptosis in methotrexate (MTX)-treated animals (Aladaileh et al., 2019a). Novel research found that FN preserved renal action in a rat model of diabetic nephropathy by hindering ROS overproduction and restoring antioxidants (Oza and Kulkarni, 2019).

However, the potential protective impact of FN against GEN-induced renal damage is yet to be studied. Therefore, this experiment sought to assess the impact of FN on GEN-induced tissue injury in the kidney, focusing on the role of Nrf2/HO-1 signaling. The findings of this study may have significant relevance for the protection of nephrotoxicity induced by GEN.

## Materials and Methods

### Animals

Twenty-four healthy adult male Wistar rats (220–250 g, 9–10 weeks of age) were used in this work to determine the protection impact of FN on GEN nephrotoxicity. The rats were accommodated in suitable cages under ideal circumstances and were supplied with balanced feed and clean water *ad libitum*. All the animal experiment handling processes in this investigation were performed following the National Institutes of Health (NIH publication No. 85-23, revised 2011). The animal experiment ethics committee of Al-Hussein Bin Talal University approved them.

### Experimental Design

After 1 week of acclimatization, the rats were allocated into four groups (*n* = 6). Group I (control): rats were orally administered with vehicles, 0.5% carboxymethyl cellulose (CMC), for 2 weeks and were intraperitoneally (i.p.) injected with normal saline during the days 8–14 (Aladaileh et al., 2019b); Group II (FN): rats orally administered FN (60 mg/kg/day, by oral route) for 14 days (Huang et al., 2017; Cai et al., 2019); Group III (GEN): rats were administered by vehicles (0.5% CMC, orally) for 2 weeks and were injected with GEN (100 mg/kg/day, i.p.) during the days 8–14 (Ahmed and Mohamed, 2019); and Group IV (FN + GEN): rats were orally administered with FN (60 mg/kg/day) for 2 weeks and were co-treated with GEN (100 mg/kg/day, i.p.) during the days 8–14. GEN (Memphis Pharmaceuticals, Egypt) and FN (Sigma, St. Louis, MO, USA) were dissociated in saline (Ahmed and Mohamed, 2019) and 0.5% CMC (Aladaileh et al., 2019a), respectively.

### Samples Collection

On day 15, animals were deeply anesthetized using ketamine and xylazine (100 mg/kg and 10 mg/kg, i.p., respectively) before performing a direct heart puncture and collecting blood samples (Alibakhshi et al., 2018). Then, immediately, both kidneys were removed, and the left kidneys were ground in cold Tris-HCl buffer (pH = 7.4) (10% w/v), centrifuged, and the clear supernatant was separated and stored in deep freezing (-80°C) for further investigations. In comparison, the right ones were settled in 10% neutral phosphate-buffered formalin for histological examination and immunohistochemistry analysis. The blood samples were left standing until complete clotting on the other side. Then, the collected samples were centrifuged, and the serum was isolated and stored in deep freezing (-80) for further biochemical analysis.

### Determination of Kidney Function Markers

According to the manufacturer's instructions, urea (Sampson et al., 1980) and creatinine (Slot, 1965) levels in serum samples were estimated by a commercial reagent kit (Spinreact, Spain) by following the protocols recommended by the manufacturer.

### Renal Oxidative Stress Markers and Antioxidants

Renal malondialdehyde (MDA) and protein carbonyl were assessed as described previously (Ohkawa et al., 1979; Levine et al., 1990), respectively. In addition, reduced glutathione (GSH) levels (Sedlak and Lindsay, 1968) and activities of superoxide dismutase (SOD) (Marklund and Marklund, 1974) and catalase (CAT) (Aebi, 1984) were assayed in the renal homogenate of all groups. According to the supplier’s instructions, renal tissue HO-1 contents were assessed using a specific ELISA kit (MyBioSource, USA) (Althunibat et al., 2022).

### Determination of Pro-inflammatory Cytokines in the Kidney

ELISA kits (R&D Systems, USA) were employed to determine interleukin 1 beta (IL-1 β), IL-6, and tumor necrosis factor-alpha (TNF-α) levels in the kidney homogenate as described previously (Al-Amarat et al., 2021).

### Histological Examination

Kidney samples were preserved in 10% formalin buffer. Post paraffin embedding, 5 μm specimens were stained with hematoxylin and eosin (H & E) for routine histopathological examination and periodic acid–Schiff (PAS) stains to illustrate more details regarding border membrane impairment, glomerular basement membrane alterations, and mucopolysaccharide deposition (Feng et al., 2017). Then, histopathological alterations in kidney cells were observed using light microscopy.

### Immunohistochemistry Analysis

Another group of deparaffinized and hydrated sections was processed by the heat-induced epitope retrieval (HIER) method using a microwave oven for immunohistochemistry analysis. Then, HIER exposed parts were immediately reacted with 0.3% H_2_O_2_ solution in methanol to block endogenous peroxidase activity. After cooling the prepared slide at room temperature, the serum was added for 20 min to block the non-specific antigen-antibody binding. Next, they were treated with anti-caspase 3, anti-BAX, anti-BCL-2, and anti-Nrf2 [all obtained from Invitrogen, CA, USA], and anti-NF-κB p65 (obtained from Santa Cruz Biotechnology, TX, USA), and kept overnight at 4°C. Post rinsing the unbound antibodies with phosphate-buffered saline, secondary antibodies were applied, followed by treatment of sections with a 3,3′-diaminobenzidine-tetrahydrochloride-H_2_O_2_ solution to induce color development. Mayer’s hematoxylin was used as a counterstain and then all sections were visualized by using light microscopy (Aladaileh et al., 2021a). Staining intensity was assessed and presented as a percentage of positive expression in 1,000 cells per eight HPF for NF-ĸB p65, caspase 3, and Bax, while Nrf2 and BCL2 immunostaining was determined through the area of positive expression using ImageJ analysis software (NIH, USA).

### Analyses of Data

All the values are presented as means ± SEM. Statistical significance between groups was obtained by GraphPad Prism 7 software (San Diego, CA, USA). For multiple comparisons, one-way ANOVA followed by Tukey’s *post-hoc* test was applied. A *p* < 0.05 was considered significant.

## Results

### FN Attenuates the GEN-Inducted Renal Damage

**FIGURE 1:**
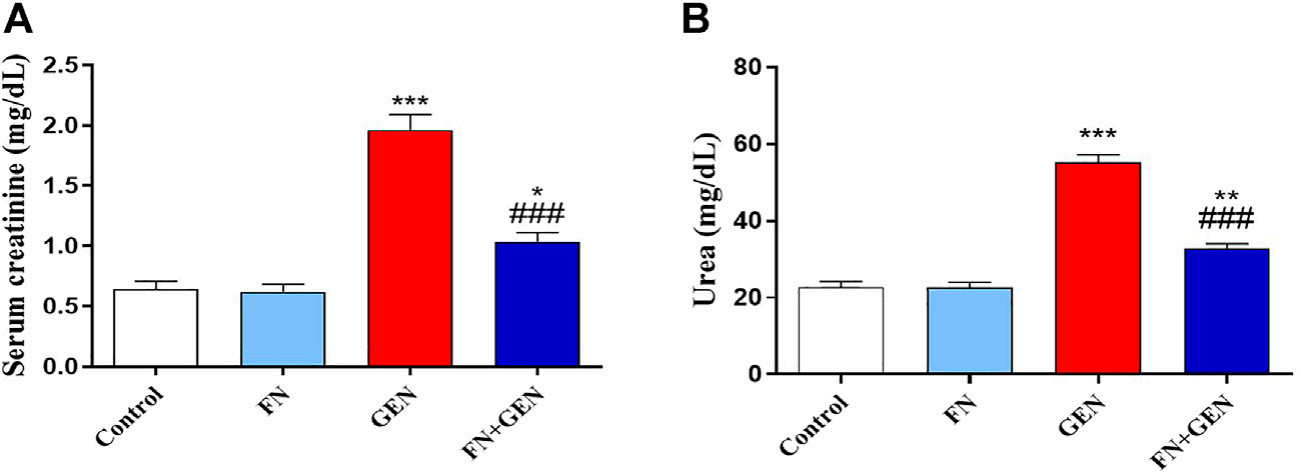
FN attenuates renal function in GEN-induced rats. FN attenuated **(A)** creatinine and **(B)** urea in the serum of GEN-intoxicated rats. Data are mean ± SEM (*n* = 6). ^∗^ indicates *p* < 0.05, ^∗∗^ indicates *p* < 0.01, and ^∗∗∗^ indicates *p* < 0.001 versus control group, while ### indicates *p* < 0.001 versus GEN group. FN: formononetin; GEN: gentamicin.

**FIGURE 2:**
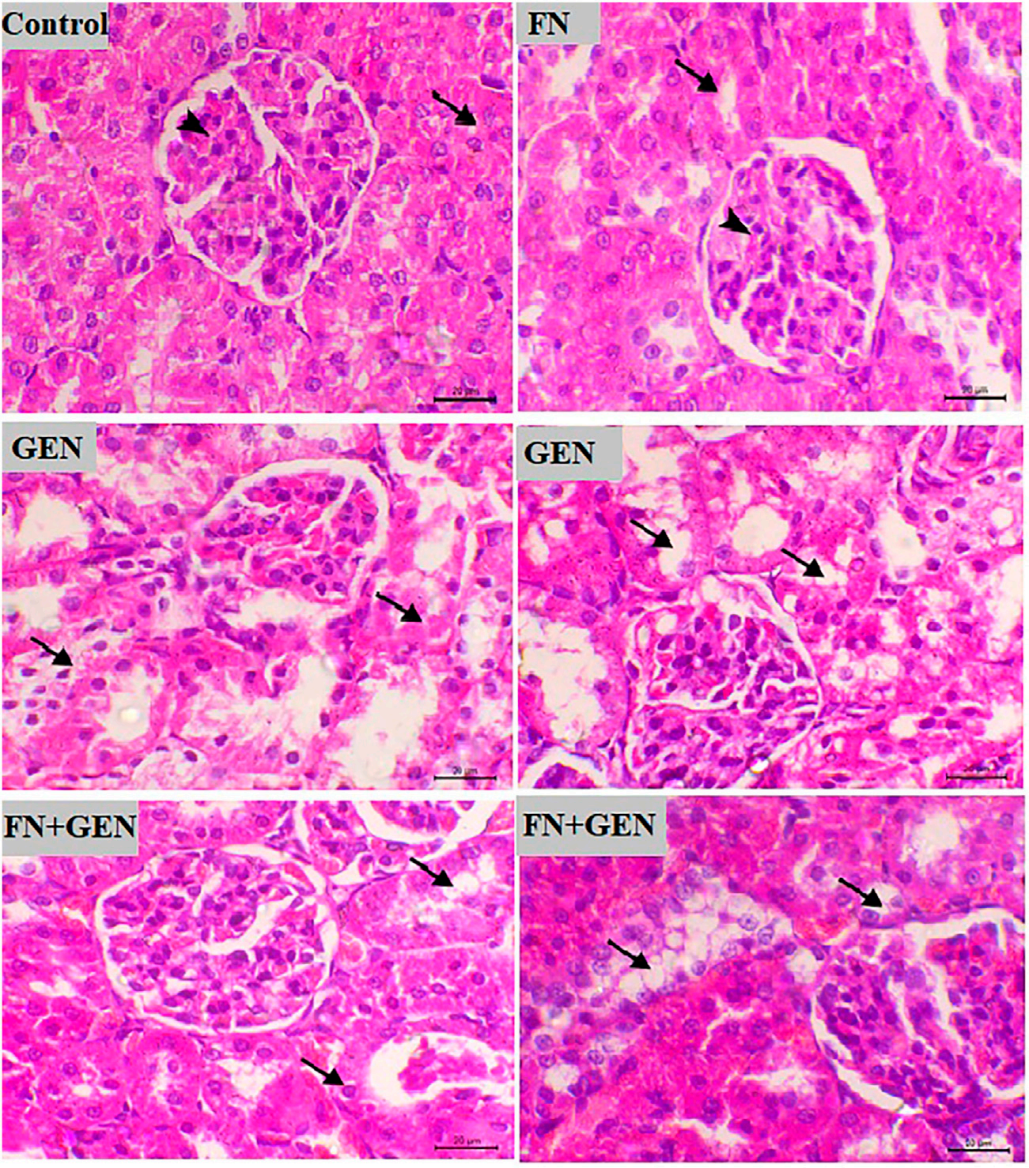
FN prevents renal destruction in GEN-administered animals. Photomicrographs of renal sections from control rats and FN-treated rats demonstrating normal histology of the glomeruli and tubules (arrowhead and arrow, respectively), GEN-treated animals demonstrating congestion of the glomerular tuft and vacuolar degeneration in the renal tubules (arrows), and GEN-administered rats pre-treated with FN showing a noticeable decrease in the vacuolar degeneration in the tubular epithelia (arrows) (H&E, X400, scale bar = 20 µm). FN: formononetin; GEN: gentamicin; and H&E: hematoxylin and eosin.

**FIGURE 3:**
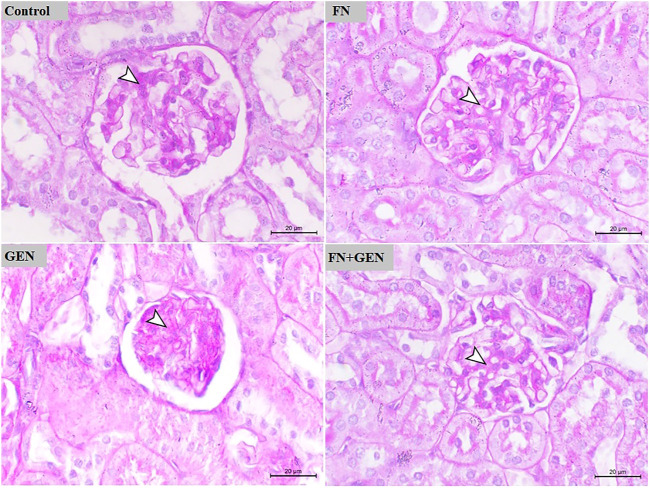
FN prevents GEN-induced histopathological damage in the kidney. Photomicrographs of renal sections of control rats and FN-treated rats demonstrating normal renal histology with very fine mucopolysaccharides on the glomerular tuft. GEN-administered rats showing glomerular atrophy associated with marked deposition of mucopolysaccharides on the glomerular tuft. GEN-administered rats pre-treated with FN showing mild deposition of mucopolysaccharides on the glomerular tuft (PAS, X400, scale bar = 20 µm). FN: formononetin; GEN: gentamicin; and PAS: periodic acid–Schiff.

The protection activity of FN against GEN nephrotoxicity was assessed by assaying biomarkers of kidney function (Figures 1A, B) and histopathological alterations (Figures 2, 3). Renal damage in GEN-induced rats was proved by the significant (*p* < 0.001) rise in creatinine (Figure 1A) and urea (Figure 1B) levels in serum. In GEN-intoxicated animals, pre-treatment with FN attenuated serum urea and creatinine (*p* < 0.001). FN did not affect these markers in normal rats.

Examination of sections from the renal tissue of normal and FN-supplied rats indicates normal tissue architecture with normal renal glomeruli and tubules. On the other hand, GEN administration resulted in degenerative vacuolar changes within the renal tubules, along with congestion of the glomerular tuft. These changes were markedly decreased when GEN-induced animals were pre-treated with FN (Figure 2).

The protection activity of FN on GEN renal toxicity was further evaluated by assessing PAS-stained kidney sections of all groups (Figure 3). PAS-stained sections of renal tissue from control and FN-supplied animals demonstrated the normal appearance of the glomeruli and kidney tubules with very fine mucopolysaccharides on the glomerular tuft. PAS-stained kidney sections of GEN-intoxicated rats revealed glomerular atrophy associated with marked deposition of mucopolysaccharides on the glomerular tuft. Pre-treatment of GEN-intoxicated rats with FN largely decreased the level of deposited mucopolysaccharides on the glomerular tuft (Figure 3).

### FN Attenuates the GEN-Induced Renal Oxidative Stress

**FIGURE 4:**
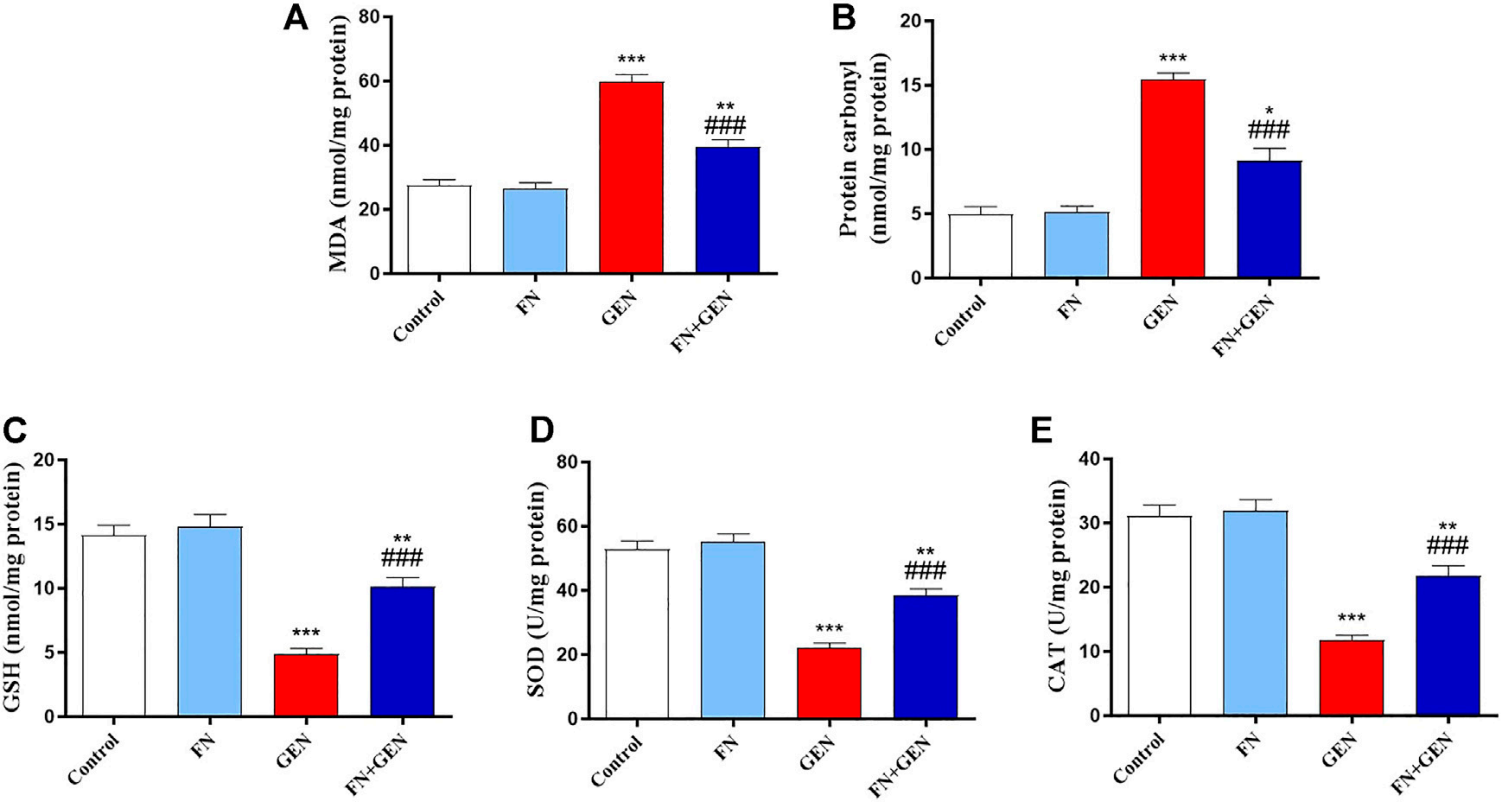
FN ameliorates kidney oxidative stress in GEN-treated rats. Pre-treatment with FN reduced renal **(A)** MDA and **(B)** protein carbonyl levels, and increased **(C)** GSH level, and activities of **(D)** SOD and **(E)** CAT in GEN-intoxicated rats. Results are mean ± SEM (*n* = 6). ^∗^ indicates *p* < 0.05, ^∗∗^ indicates *p* < 0.01, and ^∗∗∗^ indicates *p* < 0.001 versus control group, while ### indicates *p* < 0.001 versus GEN group. FN: formononetin; GEN: gentamicin; MDA: malondialdehyde; GSH: reduced glutathione; SOD: superoxide dismutase; and CAT: catalase.

Since OS is considered a key player in GEN-induced renal toxicity, we studied the impact of FN on kidney redox status. GEN supply revealed a marked elevation (*p* < 0.001) in MDA (Figure 4A) and protein carbonyl (Figure 4B). Moreover, GSH content (Figure 4C) and SOD (Figure 4D) and CAT (Figure 4E) activities in the kidney were remarkably (*p* < 0.001) decreased in GEN-intoxicated animals. All these changes in GEN-treated animals were attenuated by FN pre-treatment. FN did not significantly alter the variables mentioned earlier in normal rats.

### FN Ameliorates the GEN-Induced Inflammatory Reaction in the Renal Tissues

**FIGURE 5:**
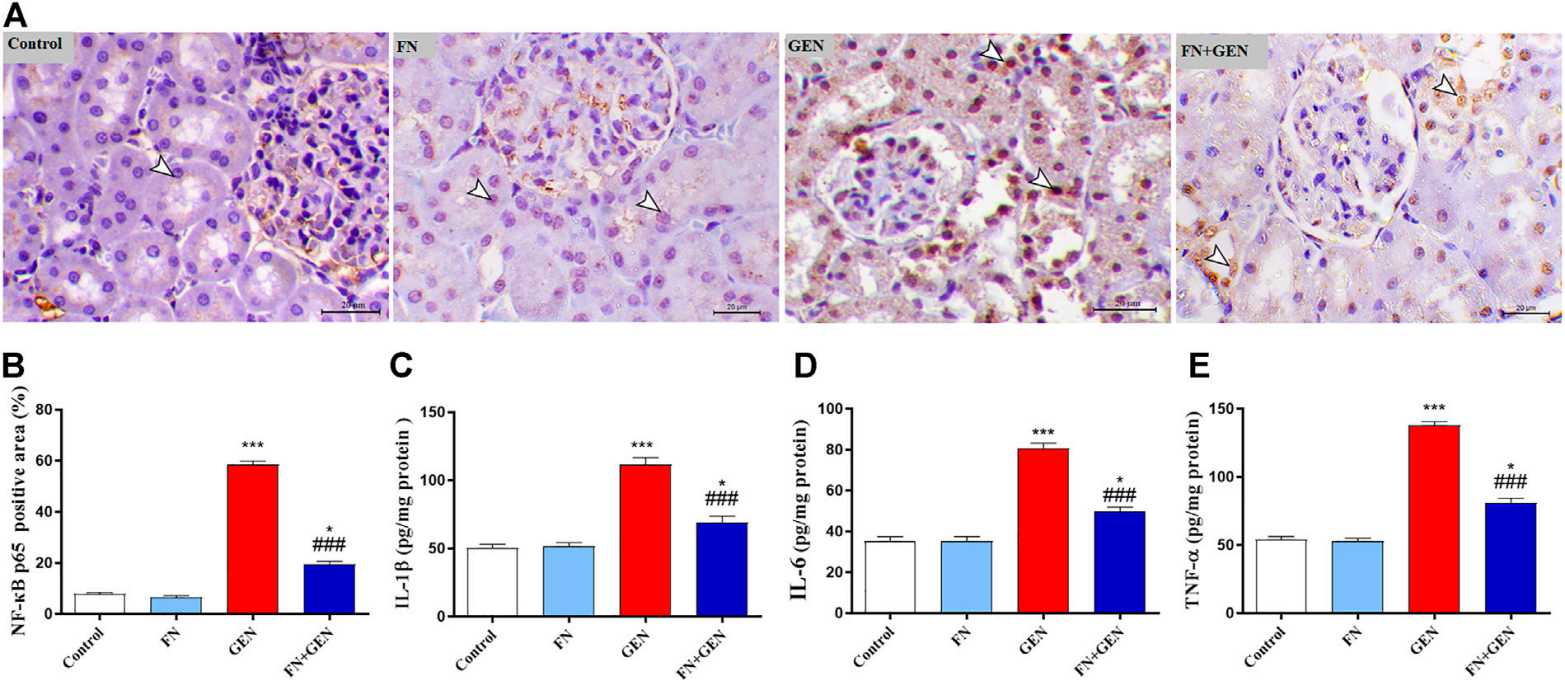
FN attenuates renal inflammatory reaction in GEN-administered rats. **(A)** Photomicrographs of renal sections from control rats and FN-treated rats demonstrating a low level of NF-κB p65 expression within the tubular epithelia (arrowheads), GEN-treated animals showing a high level of NF-κB p65 expression (cytoplasmic and nuclear) within the tubular epithelia (arrowheads), and GEN-intoxicated rats pre-treated with FN demonstrating marked decrease of NF-κB p65 expression within the tubular epithelia (arrowheads) (IHC, X200, scale bar = 20 µm). **(B)** Image analysis of NF-κB p65 immunostaining in the kidney of rats showing a significant increase in GEN-administrated rats and a significant decrease in rats treated with FN. **(C–E)** FN attenuated renal **(C)** IL-1β, **(D)** IL-6, and **(E)** TNF-α in GEN-intoxicated rats. Results are mean ± SEM (*n* = 6). ^∗^ indicates *p* < 0.05, and ^∗∗∗^ indicates *p* < 0.001 versus control group, while ### indicates *p* < 0.001 versus GEN group. FN: formononetin; GEN: gentamicin; NF-κB p65: nuclear factor kappa light chain enhancer of activated B cells p65 subunit; IL-6: interleukin 6; IL-1β: interleukin 1 β; and TNF-α: tumor necrosis factor α.

The ability of FN to suppress GEN-induced renal inflammation was evaluated through the assessment of NF-κB p65 expression and pro-inflammatory cytokine levels in the renal tissue. When contrasted with the control rats, there was a remarkable (*p* < 0.001) elevation in the degrees of NF-κB p65 (Figures 5A, B) in the renal tissue of GEN-intoxicated animals. Likewise, levels of IL-1β (Figure 5C), IL-6 (Figure 5D), and TNF-α (Figure 5E) were markedly (*p* < 0.001) elevated in the GEN-intoxicated group. The pre-treatment of GEN-intoxicated animals with FN remarkably (*p* < 0.001) downregulated renal NF-κB p65 expression (Figures 5A, B) as well as IL-1β (Figure 5C), IL-6 (Figure 5D), and TNF-α (Figure 5E) levels. FN had no effects on these inflammatory mediators in normal animals.

### FN Attenuates the GEN-Induced Renal Apoptosis

**FIGURE 6:**
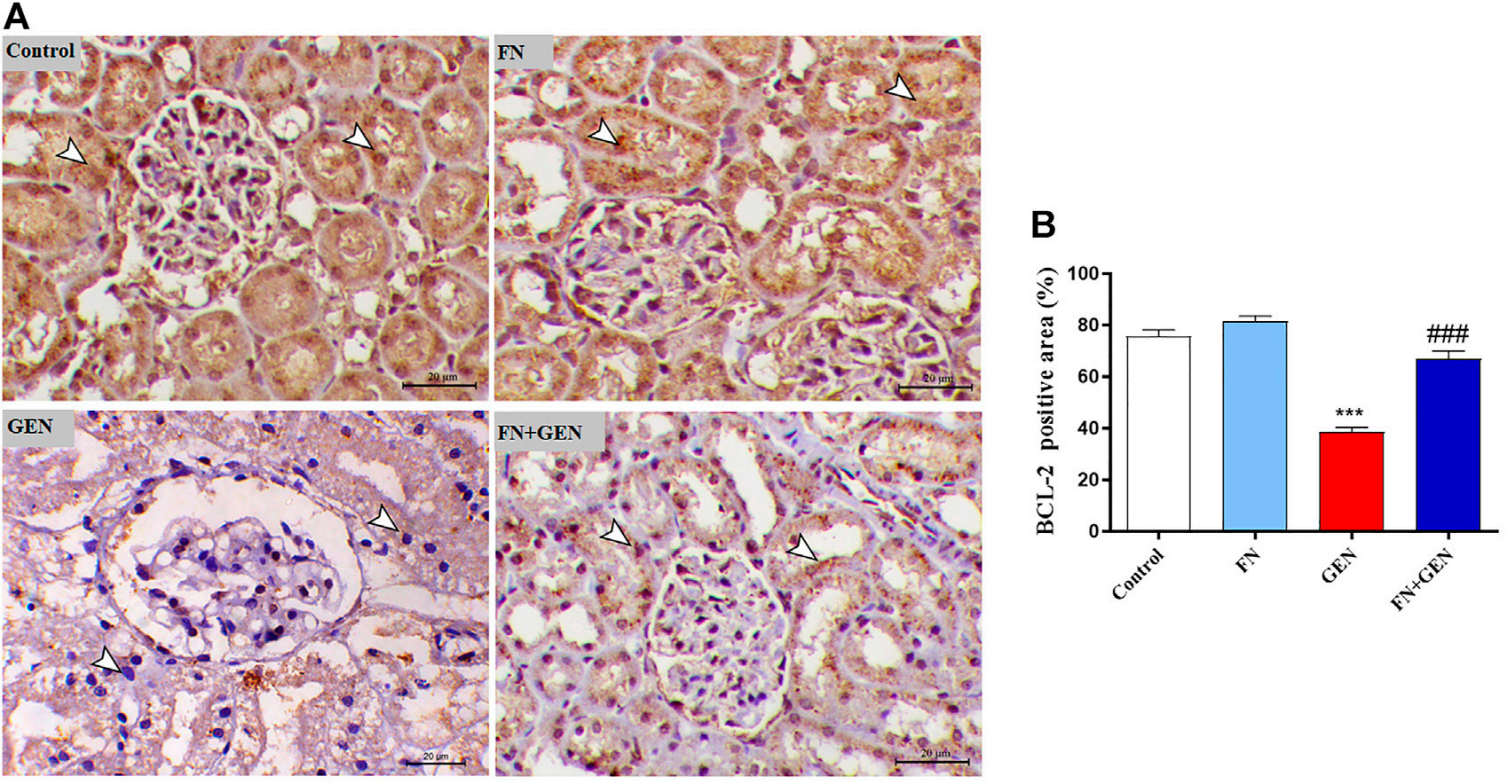
FN upregulates renal BCL-2 in GEN-intoxicated rats. **(A)** Photomicrographs of renal sections from control rats and FN-treated rats demonstrating a high level of BCL-2 expression within the tubular epithelia (arrowheads), GEN-treated rats showing a noticeable decrease in BCL-2 expression within the tubular epithelia (arrowheads), and GEN-administered rats pre-treated with FN demonstrating a noticeable increase in BCL-2 expression within the tubular epithelia (arrowheads) (IHC, X200, scale bar = 20 µm). **(B)** Image analysis of renal BCL-2 immunostaining showing a significant decrease in GEN-administrated rats and a significant increase in FN-treated rats. Results are mean ± SEM (*n* = 6). ^∗∗∗^ indicates *p* < 0.001 versus control group, and ### indicates *p* < 0.001 versus GEN group. FN: formononetin; GEN: gentamicin; and BCL-2: B-cell lymphoma 2.

**FIGURE 7:**
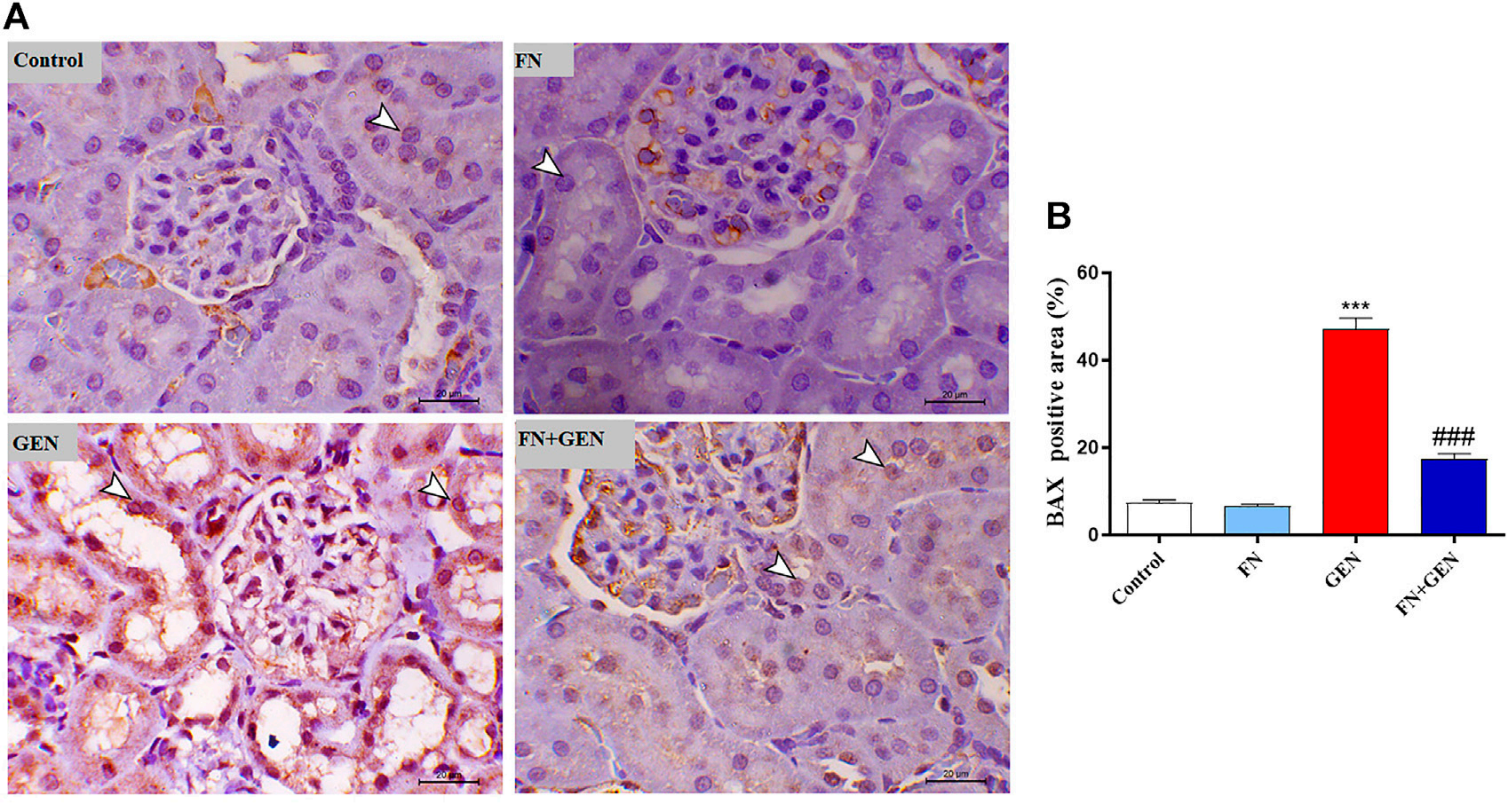
FN downregulates BAX in GEN-intoxicated kidney. **(A)** Photomicrographs of renal sections from control rats and FN-treated rats demonstrating mild expression of BAX within the renal tubular epithelium (arrowheads), GEN-treated rats animal demonstrating a noticeable increase in BAX expression within the cytoplasm and nucleus of the renal tubular epithelium (arrowheads), and GEN-administered rats pre-treated with FN demonstrating marked decrease in the expression of BAX within the renal tubular epithelium (arrowheads) (IHC, X200, scale bar = 20 µm). **(B)** Image analysis of BAX immunostaining in the kidney of rats showing a significant increase in GEN-administrated rats and a significant decrease in FN-treated animals. Results are mean ± SEM (*n* = 6). ^∗∗∗^ indicates *p* < 0.001 versus control group, and ### indicates *p* < 0.001 versus GEN group. FN: formononetin; GEN: gentamicin; and BAX: Bcl-2-associated X protein.

**FIGURE 8:**
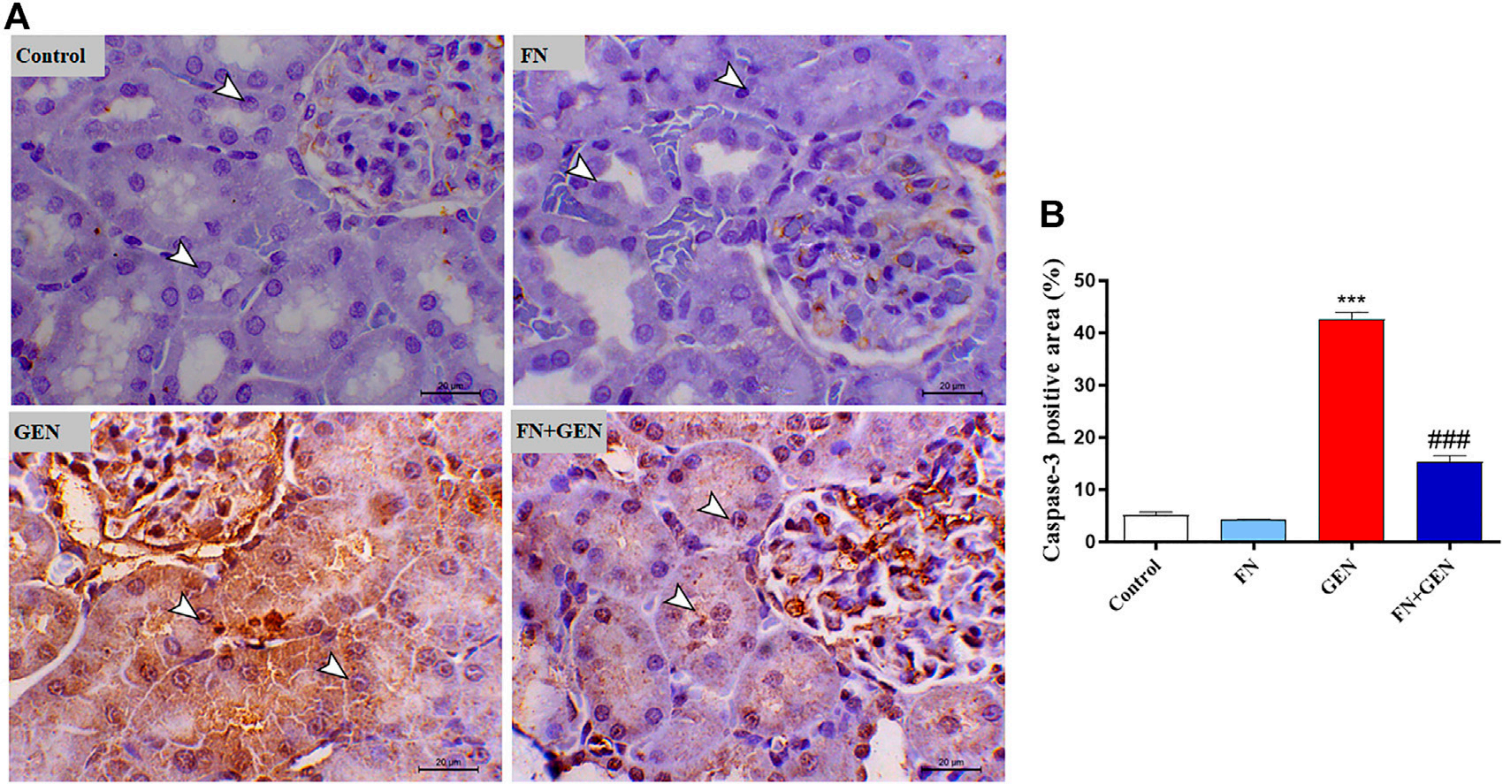
FN downregulates renal caspase-3 in GEN-intoxicated kidney. **(A)** Photomicrographs of renal sections from control rats and FN-treated rats demonstrating a low level of caspase-3 expression within the tubular epithelia (arrowheads), GEN-treated animals showing marked cytoplasmic and nuclear expression of caspase-3 within the renal tubules (arrowheads), and GEN-administered rats pre-treated with FN demonstrating a noticeable decrease in the expression of caspase-3 within the tubular epithelia (arrowheads) (IHC, X200, scale bar = 20 µm). **(B)** Image analysis of caspase-3 immunostaining in the kidney of rats showing a significant increase in GEN-administrated rats and a significant decrease in FN-treated animals. Results are mean ± SEM (*n* = 6). ∗∗∗ indicates *p* < 0.001 versus control, and ### indicates *p* < 0.001 versus GEN. FN: formononetin; GEN: gentamicin.

The persistent overproduction of ROS and inflammatory responses are major driving forces in promoting apoptosis in GEN-induced kidney levels. We evaluated renal Bcl-2, Bax, and caspase-3 expression levels. The renal tissue of GEN-treated animals revealed a marked (*p* < 0.001) reduction in Bcl-2 (Figure 6) with a concomitant increase in Bax (Figure 7) and caspase-3 (Figure 8) expression degrees as contrasted with control rats. These alterations were attenuated when GEN-induced animals were pre-treated with FN (Figures 6–8). FN alone did not significantly alter the aforementioned apoptotic proteins in normal rats.

### FN Enhances Nrf2/HO-1 Signaling in Renal Tissues of GEN-Intoxicated Rats

**FIGURE 9:**
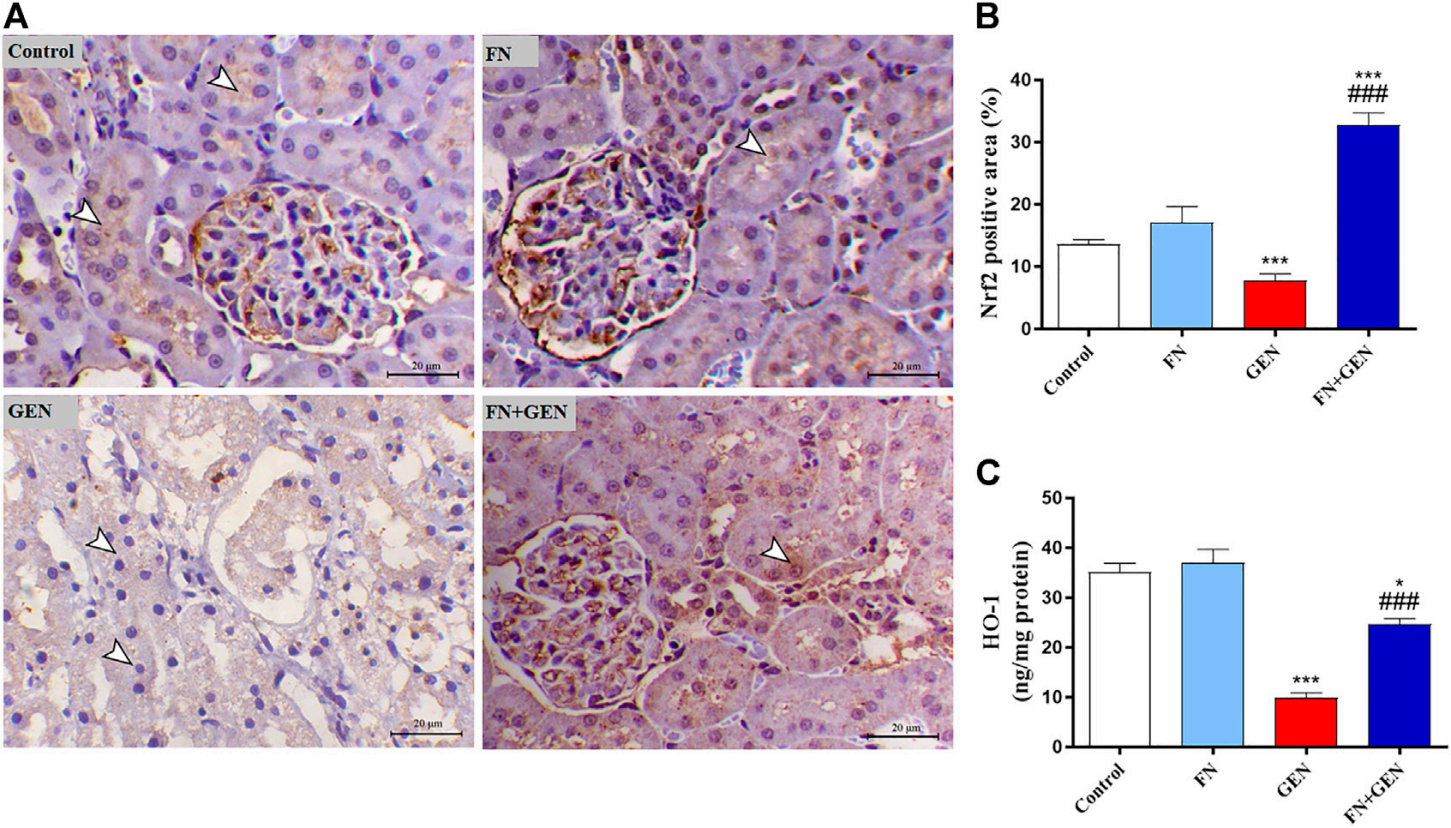
FN increases renal Nrf2/HO-1 in GEN-intoxicated animals. **(A)** Photomicrographs of renal sections from control and FN-treated rats demonstrating marked expression of Nrf2 in the tubular epithelia (arrowheads), GEN-administered animals showing decreased immunostaining of Nrf2 within the epithelial cells’ renal tubules (arrowheads), and GEN-intoxicated rats pre-treated with FN demonstrating marked increase in the expression of Nrf2 antibody within the renal tubular epithelium (arrowheads) (IHC, X200, scale bar = 20 µm). **(B)** Image analysis of Nrf2 immunostaining demonstrating significant upregulation in the kidney of GEN-intoxicated rats treated with FN. **(C)** FN increased HO-1 expression in the kidney of GEN-intoxicated rats. Results are mean ± SEM (*n* = 6). ^∗^ indicates *p* < 0.05, and ^∗∗∗^ indicates *p* < 0.001 versus control, while ### indicates *p* < 0.001 versus GEN. FN: formononetin; GEN: gentamicin; and Nrf2: nuclear factor-erythroid factor 2-related factor 2.

To further investigate the protective role of FN versus GEN renal toxicity, the expression degrees of the kidney Nrf2 and HO-1 were evaluated in GEN- and/or FN-administrated animals. As shown in Figure 9, there was a remarkable (*p* < 0.001) downregulation of Nrf2 (Figures 9A, B) and HO-1 (Figure 9C) in the kidney of GEN-intoxicated animals as compared to those of normal animals. Largely, FN pre-treatment of GEN-intoxicated rats upregulated renal Nrf2 and HO-1. Healthy animals that received FN alone showed no renal Nrf2 and HO-1 alterations.

## Discussion

Kidney damage is an abundant complication of GEN that involves the increased formation of ROS, inflammation, and caspase activation, limiting its clinical exploitation (Balakumar et al., 2010; Ahmed and Mohamed, 2019; Medić et al., 2019). FN is a naturally occurring isoflavone that has been reported to exhibit effective antioxidant and anti-inflammatory effects (Aladaileh et al., 2019a; Yi et al., 2020). Herein, we studied the effects of FN on GEN-related renal oxidative damage, inflammation, apoptosis, and consequent kidney injury. We revealed that FN attenuates oxidative tissue injury, inflammatory reaction, and apoptosis and upregulates Nrf2/HO-1 in the renal tissue of GEN-administrated animals, along with remarkable enhancement of compromised renal function.

Consistent with previous studies (Helal et al., 2018; Ahmed and Mohamed, 2019; Cao et al., 2019; Beshay et al., 2020), GEN administration induced nephrotoxicity in rats. Indeed, GEN is taken up by renal tubular cells and accumulates in the endosomes, causing phospholipidosis and disruption of their membranes. Thus, it leads to the release of GEN and other contents, including protease enzymes, eventually causing various events of cellular damage (Randjelovic et al., 2017). The GEN-induced nephrotoxicity can be defined by depletion of kidney function, divulged by elevated creatinine and urea concentrations, albuminuria, a declined glomerular filtration degree, and incidence of acute tubular necrosis (Balakumar et al., 2010; Udupa and Prakash, 2019). This is in line with our findings that revealed increased urea and creatinine levels in the serum of GEN-intoxicated rats.

Additionally, GEN administration showed a high score of microscopic renal damage, including congestion and degeneration and glomerular atrophy-associated mucopolysaccharide deposition, as illustrated in H&E- and PAS-stained sections. On the other hand, our experimental model revealed that FN exhibited a nephroprotective effect against GEN toxicity, manifested by the significant reduction in urea and creatinine concentrations in serum, in addition to remarkable improvement of histopathological changes in GEN-intoxicated rats pre-treated with FN. In accordance with our results, FN showed a potent renoprotective efficacy and alleviated kidney function in MTX-Aladaileh et al., 2019a and cisplatin-Huang et al., 2017 caused nephrotoxicity in rats.

GEN-induced nephrotoxicity has been linked to numerous pathological pathways including the existence of OS and cell death in renal tubules as well as the stimulation of inflammation in the renal cortex and medulla (Helal et al., 2018). The deleterious effect of GEN on mitochondria leads to OS by overproducing different FRs, which progresses to break the respiratory chain and decrease adenosine triphosphate (ATP) production, ultimately culminating in cell death and injury (O'Reilly et al., 2019). Herein, we found that GEN resulted in increased renal OS as revealed by elevation of renal MDA and protein carbonyl concentrations, associated with decreased CAT and SOD activities, in addition to decreased GSH level. This is in agreement with several scientific reports, which demonstrated that GEN induces overproduction of FRs, leading to oxidative cellular damage such as lipid peroxidation (LPO), DNA damage, and protein oxidation, which are implicated in the existence of GEN kidney damage (Adil et al., 2016; Beshay et al., 2020; Abdel-Fattah et al., 2021). LPO causes membrane disruption and loss of integrity and the inactivation of membrane proteins, ultimately culminating in membrane destruction (Smathers et al., 2011).

Furthermore, oxidative protein modification can disrupt the structural conformations of several proteins and damage enzyme’s active sites, including antioxidant enzymes, aggravating oxidative tissue injury (Cai and Yan, 2013). Extensive evidence indicates that OS in GEN-intoxicated kidneys is exacerbated by reducing renal antioxidant constituents, including GSH, SOD, CAT, glutathione peroxidase (GPx), and glutathione reductase (GR) (Manikandan et al., 2011; Cao et al., 2019). Therefore, suppressing OS and boosting antioxidant defenses are efficient approaches to prevent GEN nephrotoxicity. Herein, FN attenuated the GEN-induced oxidative damage in the kidneys of treated rats which was verified by reducing MDA and protein carbonyl levels with a concomitant increase in SOD and CAT activities and enhancement of GSH levels. These effects may be because of the higher antioxidant and FR scavenging potential of FN (Dutra et al., 2021; Pan et al., 2021). Consistent with our findings, FN showed antioxidant activity and thus abrogated OS-mediated nephrotoxicity induced by various drugs such as cisplatin, MTX, and cyclophosphamide (Aladaileh et al., 2019a; Aladaileh et al., 2021b; Shinde et al., 2021).

Furthermore, the inflammatory reaction is considered a main player in advancing GEN-induced kidney injury (Albino et al., 2021). Indeed, FRs are involved in activating an inflammatory mediator, NF-κB (Dos-Santos-Pereira et al., 2020). FRs produced by GEN promote NF-κB phosphorylation and dissociation from the complex (NF-κB–IB), allowing for its nuclear translocation and consequently inducing the expression of several pro-inflammatory genes as IL-6, iNOS, and TNF-α (Subramanian et al., 2015; Hassanein et al., 2021). In the same way, we tested the potential anti-inflammatory impacts of FN on GEN-evoked inflammatory markers in renal tissues. Unsurprisingly, GEN administration increased the expression of NF-κB p65, IL-1β, IL-6, and TNF-α. On the contrary, FN pre-treatment diminished inflammatory response in the kidney *via* downregulation of NF-κB p65 and its pro-inflammatory mediators. This is following a recent study conducted by our lab which proved the involvement of the anti-inflammatory capability of FN in its nephroprotective function against cyclophosphamide-induced nephrotoxicity in rats (Aladaileh et al., 2021b). Similarly, FN exhibited dose-dependent anti-inflammatory action *via* suppressing the pro-inflammatory mediators, involving IL-1β and TNF-α, as part of its renal protective mechanism against MTX-induced kidney injury (Aladaileh et al., 2019a). Furthermore, FN was found to protect liver tissues of the murine model versus concanavalin-A-caused autoimmune hepatitis *via* suppressing NF-κB signaling and the inflammatory process (Liu et al., 2021).

Notably, the tissue oxidative damage and inflammation in GEN nephrotoxicity are closely interrelated, eventually culminating in mitochondrial dysfunction and tissue apoptosis (Balakumar et al., 2010; He et al., 2015; Promsan et al., 2016; Sepand et al., 2016; Ahmed and Mohamed, 2019; Medić et al., 2019). Herein, GEN-intoxicated rats showed tissue apoptosis manifested by elevated Bax and caspase-3 and lowered Bcl-2. Indeed, apoptosis in GEN-induced animals is thought to be elicited *via* increased ROS and pro-inflammatory mediators, resulting in pro-apoptotic factors and diminishing anti-apoptosis factors (Morales et al., 2010; Promsan et al., 2016; Sepand et al., 2016). Thus, attenuation of OS and pro-inflammatory pathways may prevent GEN nephrotoxicity and apoptosis. Herein, FN protected against GEN-induced renal apoptosis in rats as evidenced by stimulated expression of Bcl-2 and lowered expression of Bax and caspase-3. Consistently, FN attenuated apoptosis *via* downregulating Bax and caspase-3 and upregulating Bcl-2 in rats' model of MTX-Huang et al., 2017- and cisplatin-Huang et al., 2017-induced nephrotoxicity. The ability of FN to prevent GEN-induced renal apoptosis could be attributed to its potential inhibitory effects on ROS and pro-inflammatory cytokine formation.

To further understand the potential underlying process of how FN boosted the antioxidant defenses and protected against GEN-caused tissue injury, we estimated the possible action of FN on Nrf2/HO-1 signaling in the kidney. Multiple lines of evidence indicate that Nrf2 is a key player in regulating the expression of a large spectrum of antioxidant and detoxifying genes and enhancing cellular defense against oxidative damage (Mahmoud et al., 2017a; Mahmoud et al., 2017b). Under normal redox status, Kelch-like ECH-accompanied protein 1 (Keap1) binds to Nrf2 and mediates its destruction by the ubiquitin-dependent proteasomal degradation pathway (Klaassen and Reisman, 2010; Satta et al., 2017). Increased intracellular oxidant agents, on the other hand, disrupt the sequestration of Nrf2 by Keap1, facilitating its nuclear translocation, where it binds to the antioxidant reaction element (ARE) to stimulate the gene expression of several enzymes, such as glutathione S-transferase (GST), NADPH-quinone oxidoreductase 1 (NQO1), and HO-1 (Loboda et al., 2016). In this study, GEN lowered Nrf2 signaling in the kidney, as proved by the suppressed Nrf2 and HO-1 levels. Consistently, several studies showed decreased Nrf2 and HO-1 levels in the renal tissue of GEN-handled animals (He et al., 2015; Wang et al., 2020).

Also, accumulating expertise shows that a large spectrum of natural materials that stimulate the Nrf2/HO-1 signaling pathway provided an additional protective strategy against GEN nephrotoxicity (He et al., 2015; Promsan et al., 2016). Herein, FN effectively increased Nrf2/HO-1 signaling in GEN-treated animals. Accordingly, these findings supported previous studies where FN treatment upregulated renal Nrf2/HO-1 signaling in MTX-administrated animals (Aladaileh et al., 2019a) and cisplatin nephrotoxicity (Hao et al., 2021). Moreover, several reports indicated that Nrf2 is considered a key player in protecting against oxidative damage and regulating inflammatory and cell death pathways (Wardyn et al., 2015; Huang et al., 2016). This assumption is supported by a previously published report which showed that deletion of Nrf2 is associated with enhanced inflammation while its upregulation reduced pro-inflammatory cytokines regulated by NF-ĸB (Wardyn et al., 2015). Moreover, activated Nrf2 and HO-1 regulate the inflammatory cascade through inhibition of NFκB signaling and activating anti-inflammatory cytokines (Wardyn et al., 2015; Ahmed et al., 2017). Also, activation of Nrf2 by FN suppressed the inflammatory response of proximal tubule epithelial cells in cisplatin nephrotoxicity in animals (Hao et al., 2021). It inhibited intracephalic inflammatory response in brain tissue of a rat model of traumatic brain injury (Li et al., 2014). In addition, FN-mediated Nrf2 upregulation prevented renal apoptosis by lowering BAX and caspase-3 levels in rhabdomyolysis-induced renal damage in rats (Satta et al., 2017). Taken together, FN can effectively prevent GEN-caused kidney destruction, possibly *via* upregulation of Nrf2/HO-1 signaling.

## Limitation

This study demonstrates the renoprotective effects of FN on GEN-induced nephrotoxicity; however, it has some limitations. While our study clearly showed upregulation and downregulation of some proteins as indicated by immunohistochemistry, we did not validate it by RT-qPCR to show the relationship between mRNA and protein levels.

## Conclusion

Our findings demonstrate that FN may have promising therapeutic potential against GEN-induced nephrotoxicity *in vivo*. FN ameliorated kidney function markers, prevented histopathological alterations, alleviated oxidative tissue damage, and boosted antioxidants in the kidney of GEN-administrated rats. In addition, FN downregulated GEN-induced NF-κB activation, pro-inflammatory cytokine release, and apoptotic cell death in the kidney. These beneficial effects were associated with the upregulation of Nrf2/HO-1 signaling in the kidney of GEN-administrated rats. Therefore, FN might represent a promising approach against the renal complications of GEN, pending further investigations in upcoming studies.

## Data Availability

The article contains all data supporting the reported results.

## Abbreviations

ARE, antioxidant response element; BAX, Bcl-2-associated X protein; BCL-2, B-cell lymphoma 2; CAT, catalase; CMC, carboxymethyl cellulose; FN, formononetin; GEN, gentamicin; GSH, reduced glutathione; GPx, glutathione peroxidase; GR, glutathione reductase; GST, glutathione S-transferase; HIER, heat-induced epitope retrieval; HO-1, heme oxygenase 1; IHC, immunohistochemistry; IL-1β, interleukin-1β; IL-6, interleukin-6; iNOS, inducible nitric oxide synthase; LPO, lipid peroxidation; MDA, malondialdehyde; MTX, methotrexate; NF-κB, nuclear factor kappa B; NO, nitric oxide; Nrf2, nuclear factor erythroid 2-related factor 2; OS, oxidative stress; PAS, periodic acid–Schiff; RNS, reactive nitrogen species; ROS, reactive oxygen species; SOD, superoxide dismutase; and TNF-α, tumor necrosis factor-alpha.
